# Trancriptome data mining in combination with co-expression network analysis identifies the functional modules and critical regulators in *Hordeum vulgare* L. in response to cold stress

**DOI:** 10.1016/j.bbrep.2023.101620

**Published:** 2023-12-20

**Authors:** Bahman Panahi, Ali Shahi

**Affiliations:** a Department of Genomics, Branch for Northwest & West Region, Agricultural Biotechnology Research Institute of Iran (ABRII), Agricultural Research, Education and Extension Organization (AREEO), Tabriz, Iran; b Faculty of Agriculture (Meshgin-Shahr Campus), University of Mohaghegh Ardabili, Ardabil, Iran

**Keywords:** Barley, Coexpression, Cold, Hub, Kinase, Regulatory network

## Abstract

Cold stress, as an abiotic stress, is one of the most limiting factors which pose a great threat to the plant's productivity. To understand the transcriptional regulation and connectivity pattern of genes involved in barley cold stress responses, co-expression network analysis was performed based on the global transcriptome profiling. The microarray datasets related to cold stress treatments were retrieved from the Gene Expression Omnibus (GEO) and Array express databases. Four microarray datasets related to cold stress-responsive transcriptome in barley were included in our study. Gene co-expression analysis was constructed using WGCNA method. Module-Trait Relationships (MTR) analysis and hub genes determination and validation were carried out. Finally, transcription factor and kinase regulatory networks were Inferred using machine learning algorithm. The co-expression modules were determined using beta index = 10. In total 13 co-expressed modules were identified with an average size of 153 genes. Functional enrichment based on gene ontology (GO) showed that each of the stress related significant modules were enriched in different biological processes. Annotation of significant modules identifies some TFs and Kinases such as ethylene-responsive transcription factor 1-like, transcription factor PCL1-like, transcription factor MYC2, WRKY, serine/threonine-protein kinase PBL7, and receptor-like protein kinase At2g42960 were contributed in barley cold stress response. Our analysis highlighted the functional importance of ABA signaling pathway, ROS signaling, defensive and protective proteins, degrading protein, Ca2^+^ related signaling, ribosome-mediated translation and etc. in responding of barley to cold stress condition. The current findings add substantially to our understanding of the cold responsive underlying mechanism of barley which can serve in future studies and breeding programs.

## Highlights

• Annotation of significant modules identifies some TFs and Kinases were contributed in barley cold stress response.
• Our analysis highlighted the functional importance of ABA signaling pathway, ROS signaling, defensive and protective proteins, degrading protein, Ca2^+^ related signaling, ribosome-mediated translation and etc. in responding of barley to cold stress condition.
• The current findings add substantially to our understanding of the cold responsive underlying mechanism of barley which can serve in future studies and breeding programs.

## 1 Introduction

Cold stress, as an abiotic stress, is one of the most limiting factors which pose a great threat to the plants survival, growth and seed productivity [1]. According to the report of the Intergovernmental Panel on Climate Change (IPCC), cold stress is frequently occurred in the tropical and subtropical regions, leading the severely limitation of the crop plants distribution [2].

Cold stress has significant effects on the physiological, biochemical, and molecular parameters in plants. Among different physiological responses, photosynthesis machinery is severely impeded under cold stress condition, leading the inhibition of the biosynthesis of chlorophyll, reduction of the enzymatic activity associated with photosynthesis and photochemical efficiency and overall photosynthesis rate [3]. Destruction of the cellular membrane structure and destabilisation of nucleic acid structure are other physiological responses of plant to cold stress circumstances [3,4]. It has been demonstrated that the cold stress induces the reactive oxygen species (ROS) accumulation causes severe oxidative damage to DNA and proteins, damage the membrane structure, and decrease the fluidity of the cell membrane [5]. To diminish the oxidative damage under cold stress condition, plant cells increases the levels and activity of the ROS scavenging enzymes (Wang et al., 2020).

On the other hand, change of the gene expression pattern induces multi-levels responses that increase plant cell tolerance under cold stress conditions. Transcriptome and genomic analyses have shown that the expression pattern of thousands of genes which were related to the carbohydrates metabolisms, signal transduction, and regulatory systems were varied and are potential importance in coordinating cold tolerance with growth and development [[6], [7], [8], [9]]. The expression of a gene is determined by a number of transcription factors as a master gene expression regulator. As many transcription factor (TF) genes were found among the stress-inducible genes, it has been hypothesized that there are different transcriptional regulatory mechanisms in the cold stress signal transduction pathways. The C-repeat-binding factor/dehydration-responsive element-binding factor 1 (CBF/DREB1) family is among the most important family of TFs associated with the cold stress response of plants. Inducer of CBF expression 1 (ICE1), as a key regulator of CBF genes, constitute the ICE1-CBF signaling pathway, which has an important role in the tolerance of plants under cold stress conditions [7]. Moreover, WRKYs, MYBs, and ZAT12 were found to exhibit cold-induced properties ([10,11]; An et al., 2018; [12]). Other actors in plant cold response scenario are kinase (receptor kinase, protein kinase/phosphatase), ROS, hormones, and non-coding RNAs [13,14]

Due to the extensive application of global expression profiling, the amounts of deposited transcriptome data are exponentially increased. Therefore, generalization of the stress underlying mechanism is essential to provide meaningful and precise biological conclusions. On the other hand, most of the studies focus only on the identification of differentially expressed genes and connectivity and systems level analysis were not well considered [5,15,16]. It has been proven that the co-expression based network analysis in the context of the transcriptome information provides valuable knowledge regarding the systems level behavior of differentially expressed genes under a specific condition, which cannot be detected by standard transcriptome analysis [[17], [18], [19], [20]]

Barley (*Hordeum vulgare*) is one of the most important cereal crops in the world with major usage in food and animal feed and ranked fourth after wheat, rice, and corn in terms of planting area and average yield as reported by faostat (faostat.fao.org). Recent advances in characterizing the barley genome and gene expression profiling using high throughput approach provides a promising chance to identify the genes and underlying mechanisms, which is a vital step for developing barely able to cope with the cold stress [21].

To understand the transcriptional regulation and connectivity pattern of genes involved in cold stress responses, co-expression network analysis were performed. Moreover, underlying key hubs, pathways and regulatory TFs of barely under cold stress condition were dissected.

## 2 Methods and material

### 2.1 Eligible transcriptome data selection

This meta-analysis was performed by selecting publications dealing with responses to cold stress in barley on the Google Scholar database (http://scholar.google.com). The keywords used were ‘cold stress’, ‘chilling stress’, ‘barley, and ‘transcriptomics’. The database survey was carried out in order to select the manuscripts containing transcriptomic data obtained from experiments performed in comparable conditions. In particular, studies conducted at the same stress time points (0 h and 24 h of chilling treatment) and under similar temperatures (4–6 °C) were considered. The criteria applied during the literature investigation led to the selection of four studies performed on barley.

The transcrriptome datasets related to cold stress treatments. Four microarray datasets related to cold stress-responsive transcriptome in barley were retrieved from the Gene Expression Omnibus (GEO) and Array express databases and included for further study. The first dataset (GSE10329) had 12 biological samples from barley grown under cold and control conditions. Plants were grown at 20 °C for seven days and subject to a symmetrical cycle of acclimation, cold, freeze-thaw, and deacclimation. Cold stress began by decreasing the temperature overnight from 20 °C to 4 °C at a rate of 1.3 °C ° h^−1^ and maintaining temperatures of 4 °C in the day and 2 °C at night for 5 days. The second dataset (GSE10332) covers microarray data of barley with cold treatment and control with three replicates. The third dataset (GSE27821) contains 12 samples of global transcriptom profiling of barley in response to cold treatments. The fourth data set (GSE27822) which had 6 samples surveyed the transcriptomic changes induced by cold stress and control conditions with three replicates. In the entire microarray datasets which were used in current study Affymetrix Barley Genome Array (GPL1340) were harnessed for expression profiling.

### 2.2 Microarray data analysis

Quality control assessment of microarray data is crucial to avoid the negative impacts and bias in downstream analysis, by identify potential low-quality arrays and impairing statistical and biological significance. Assessment of microarray quality in current study was performed using arrayQualityMetrics (Kauffmann et al., 2006) Bioconductor package. Moreover, additional QC/QA analysis was carried out to provide the intensity distribution of each array in comparison with other arrays. Data sets with acceptable quality were subjected for normalization. The background correction, data normalization, and probe summarization using default parameters in the limma package (Diboun et al., 2006). Control probes were removed before quantile normalization in R using the quantiles function. The probesets were mapped to Entrez gene IDs according to the array annotation table provided by NCBI GEO. The genes labeled with more than one probeset were filtered by their relative standard deviation (RSD). The probeset with the highest RSD was retained, which guaranteed the useful information. Before direct merging of normalized data sets to get common genes, ComBat method implemented in the sva package [22] were used to batch-adjust the expression data of the merged dataset.

### 2.3 Network construction

Because the gene co-expression analysis is sensitive to outliers, the distance-based adjacency metrics of samples was calculated and samples with a standardized connectivity less than −2.5 were excluded. Moreover, samples which contain the genes with higher than 50 % missing entries and zero variance were removed from network construction. In the next step, the correlation of common genes was determined with Pearson's correlation algorithm. Then, created similarity matrix was transformed into an adjacency matrix using the following formula:

$$a_{ij}={\left(0.5*\left(1+\text{cor}\left(i,j\right)\right)\right)}^{ᵝ}$$

Where $a_{ij}$ represent the adjacencies between genes as a connection strengths index.

The adjacency was transformed into a topological overlap matrix (TOM) and corresponding dissimilarity matrix (1−TOM) using the following equation

$${TOM}_{i,j}=\frac{\sum_{u}a_{iu}\;a_{uj}+a_{ij}}{\min\left(k_{i},k_{j}\right)+1-a_{ij}},K_{i}=\sum_{u}a_{iu}$$

Where row index u (u=1,…,m) represents the sample measurements.

Finally, average linkage hierarchical clustering analysis was performed by the topological overlap based dissimilarity matrix as input, and modules were determined by a dynamic hybrid tree cutting algorithm implemented in the WGCNA R package.

### 2.4 Module-trait relationships (MTR) analysis

To identify significant highly-correlated modules with cold stress responsive mechanism in barley, informative models related to stress responsive, transcriptome profiling of cold treated and control samples of barley were used for module-trait relationships (MTRs) analysis. In order to identify the modules which were significantly related to stress responsive mechanisms, the correlation between the stress status (control/stress) and module eigengenes was taken using Pearson correlation coefficient.

### 2.5 Determination of hub genes

In complex biological networks such as a response to a different physiological condition, the genes with the highest degree of connectivity in the systems level networks were considered essential genes with significant importance related to the studied biological process. Based on this fact, hub genes in modules with significant correlation with cold stress responsive mechanisms were determined according to module membership (MM) and module eigengene (ME) using following formula.

$$MM\left(i\right)=cor\left(x_{i},ME\right)$$

MM is defined to estimate the importance of each gene in a module. The larger the absolute value of MM (i), the more similar the gene i is to the eigengene of a module, i.e. the more important the gene i is in a module. Determined hub genes were further validated using leave-one-out cross-validation (LOOCV) algorithm.

### 2.6 Inferring transcription factor and kinase regulatory networks

Transcription factor (TF) annotations for the barley genome were compiled based on proteome homology studies compiled as described by Ref. [23]. We obtained the protein IDs of predicted TFs from Ref. [24]. In the next step, the gene regulatory networks between top hub genes of significant modules and TFs were inferred using ensemble of trees algorithm implemented in GENIE3 R package.

### 2.7 Functional enrichment analysis

To survey of the functional impacts of significant modules, enrichment analysis was performed based on gene ontology (GO) and the Kyoto encyclopedia of genes and genome (KEGG) pathway using ClueGO plugin of cytoscape software by setting P-value <0.05 as a cut-off criterion.

### 2.8 Leave-one-out cross validation

To validate the results, Leave-one-out cross validation (LOOCV) was employed on expression values of hub genes to evaluate the efficiency of hub genes to differentiate between samples under cold stress and control conditions.

## 3 Results

### 3.1 Network construction and module determination

**Fig. 1:**
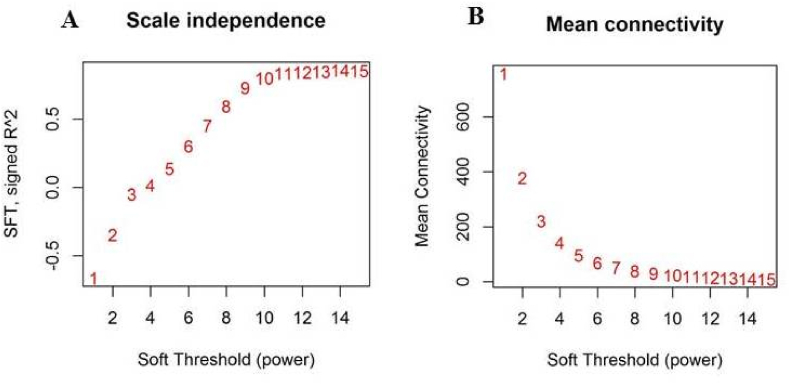
The relationship of Soft Threshold (power) with Scale Free Topology (A) and Mean Connectivity (B).

Collected eligible raw microarray sets contained 64 samples of which 34 were related to stress treated samples and 30 samples were among the control samples. After filtering of genes with low variance, the final matrix contain 2144 genes were used for co-expression network construction and further analysis. As mentioned in the method and material section, to construct the reliable network, outliers must be removed. Distance-based adjacency analysis showed that four samples of stress treated groups had a standard connectivity score lower than −2.5, were removed and the remaining samples (60 samples) included in weighted co-expression network construction (Fig. 1).

**Fig. 2:**
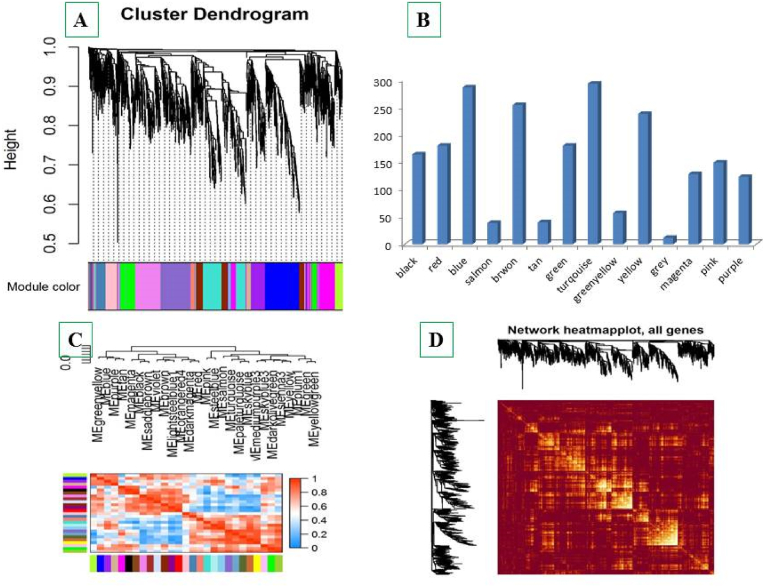
Weighted gene co-expression network analyses of stress responses genes in barley. (A) Determined functional modules based on hierarchical cluster tree of the common genes. (B) The sizes of determined modules based on the number of involved genes. (C) The module Eigen gene adjacency estimated by hierarchical clustering and shown by heat map. (B) Topological Overlap Matrix (TOM) value among the proteins of the network delimited in modules by the dynamic method.

Soft-Threshold (power) was tested between 1 and 15 and results showed that in the power = 10, the Scale Free Topology fitting index (R2) was higher than 0.8 and connectivity mean became stable (Fig. 1A and B). Therefore, the co-expression modules were determined using beta index = 10. In total 13 co-expressed modules were identified with an average size of 153 genes (Fig. 2A). Of which, turquoise and salmon modules with a size of 293 and 39 genes were the largest and smallest modules, respectively (Fig. 2B). The genes which were not shown any correlation were allocated to the gray module with 12 genes. Moreover, the heat map shows the topological overlap matrix (TOM) value among the nodes of the network delimited in modules by the dynamic method (Fig. 2C). Yellow and progressively red colors indicate the low and higher TOM values, respectively. Expression profiles of each module were summarized as the eigenvector correlated to the first main component of the expression matrix using the module eigengene (Fig. 2 D.)

### 3.2 MTR analysis

**Fig. 3:**
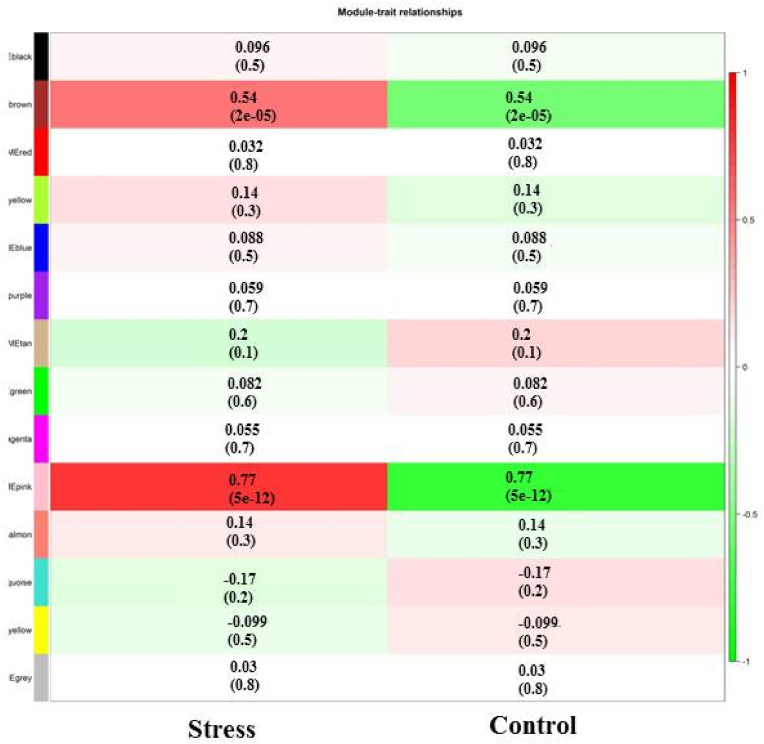
Module-Trait relationships analysis of co-expressed modules in response to cold stress condition.

To determine the significant associated module relates to cold stress responsive mechanism, MTR analysis was performed. Results showed that the two brown and pink modules with 254 and 145 genes, respectively, significantly correlated (0.77 and 0.54 % correlation for pink and brown, respectively) with cold responsive mechanisms (Fig. 3). These modules were considered as most informative modules and further functional and regulatory network inferring were performed on these two modules.

### 3.3 Functional enrichment analysis

Functional enrichment analysis of significant modules was performed to shed light on the biological performance of underlying genes in the network context. Functional enrichment based on gene ontology (GO) showed that each of the stress related significant modules were enriched in different biological processes. For example, GO terms such as “Proteasomal ubiquitin-independent protein catabolic process” (GO: 0010499), “Reactive oxygen species metabolic process” (GO: 0072593), “Regulation of stomatal closure” (GO: 0090333), “Response to abiotic stimulus” (GO: 0009628), and “Abscisic acid-activated signaling pathway” (GO: 0009738) were significantly enriched in the brown module (Table 1). It is whilst that the most significant enriched GO term in the biological process category of the pink module were “Transcription by RNA polymerase II” (GO: 0006366), “Translation” (GO: 0006412), “Primary metabolic process” (GO: 0044238), “Beta-alanine metabolic process” (GO: 0019482), and “Response to carbohydrate” (GO: 0009743) (Table 1). The significantly enriched KEEG pathways of the significant modules related to cold response in barley were presented in Table 2. The most significant KEGG pathway in the brown module was “Proteasome” (ath03050) and “Metabolic pathways” (ath01100) (Table 1). It is whilst, significantly KEGG enriched pathways in the pink models were “Proteasome” (ath03050), “Ribosome” (ath03010), and “Ribosome biogenesis” (ath03008) (Table 1).

**Table 1:** Significantly enriched GO terms in brown and pink modules.

Modules	GO ID	Description	FDR
Brown	GO:0010499	Proteasomal ubiquitin-independent protein catabolic process	0.0023
Brown	GO:0051603	Proteolysis involved in cellular protein catabolic process	0.005
Brown	GO:0006511	Ubiquitin-dependent protein catabolic process	0.0081
Brown	GO:0072593	Reactive oxygen species metabolic process	0.0095
Brown	GO:1901701	Cellular response to oxygen-containing compound	0.0095
Brown	GO:0090333	Regulation of stomatal closure	0.0176
Brown	GO:0000398	mRNA splicing, via spliceosome	0.0227
Brown	GO:0009056	Catabolic process	0.0245
Brown	GO:0009628	Response to abiotic stimulus	0.0245
Brown	GO:0042221	Response to chemical	0.0245
Brown	GO:0000303	Response to superoxide	0.0278
Brown	GO:0051170	Import into nucleus	0.0278
Brown	GO:1903409	Reactive oxygen species biosynthetic process	0.0278
Brown	GO:0071470	Cellular response to osmotic stress	0.0309
Brown	GO:1901700	Response to oxygen-containing compound	0.0309
Brown	GO:0036228	Protein localization to nuclear inner membrane	0.0356
Brown	GO:0006508	Proteolysis	0.0367
Brown	GO:0006807	Nitrogen compound metabolic process	0.0367
Brown	GO:0010119	Regulation of stomatal movement	0.0367
Brown	GO:0009738	Abscisic acid-activated signaling pathway	0.0438
Brown	GO:0008380	RNA splicing	0.0464
Pink	GO:0006412	Translation	0.00085
Pink	GO:0006518	Peptide metabolic process	0.00085
Pink	GO:0043604	Amide biosynthetic process	0.00085
Pink	GO:1902531	Regulation of intracellular signal transduction	0.0049
Pink	GO:0009743	Response to carbohydrate	0.0076
Pink	GO:0006366	Transcription by rna polymerase ii	0.0148
Pink	GO:0044238	Primary metabolic process	0.0148
Pink	GO:0050896	Response to stimulus	0.0148
Pink	GO:0034641	Cellular nitrogen compound metabolic process	0.0163
Pink	GO:0042254	Ribosome biogenesis	0.0163
Pink	GO:0019482	Beta-alanine metabolic process	0.0381
Pink	GO:0072344	Rescue of stalled ribosome	0.0381
Pink	GO:0051246	Regulation of protein metabolic process	0.0429

**Table 2:** Significantly enriched KEGG pathways in two brown and pink modules.

Modules	KEGG ID	Description	FDR
Brown	ath03050	Proteasome	0.00000092
Brown	ath01100	Metabolic pathways	0.0499
Pink	ath03050	Proteasome	2.98E-05
Pink	ath03010	Ribosome	0.00046
Pink	ath03008	Ribosome biogenesis	0.0251

### 3.4 TFs, kinase and hub genes in significant modules

Annotation of significant modules identifies some TFs and Kinases which are contributed in barley cold stress responsive mechanisms. As shown in Table 3, TFs such as ethylene-responsive transcription factor 1-like, transcription factor PCL1-like, transcription factor MYC2, heat stress transcription factor A-4b-like, and MADS-box transcription factor 16 were identified in brown module. It is whilst that the WRKY were the most abundant TFs in the pink module.

**Table 3:** Identified transcription factor (TFs) in two brown and pink modules.

Module	Uniprot ID	TFs
Brown	A0A8I6XQF9	ethylene-responsive transcription factor 1-like (ERF1)
Brown	E3VXB6	ethylene-responsive transcription factor 1-like (ERF1A)
Brown	F2CYY5	ethylene-responsive transcription factor 1-like (ERF1A)
Brown	F2D0U8	transcription factor PCL1-like
Brown	F2D1Q9	transcription factor MYC2
Brown	F2D332	transcription factor PCF6-like
Brown	F2D3K2	heat stress transcription factor A-2c-like
Brown	F2DMG4	transcription factor VIP1-like
Brown	F2DMM4	myb family transcription factor IPN2-like
Brown	F2E4P7	heat stress transcription factor A-4b-like
Brown	F2E8K7	ethylene-responsive transcription factor 1-like
Brown	F4ZC62	heat stress transcription factor A-4b-like
Brown	Q6QHI3	MADS-box transcription factor 16
Brown	S5UFU6	transcription factor PCL1-like
Pink	A0A8I6XNX2	WRKY transcription factor 65
Pink	A0A8I7B6Z9	WRKY transcription factor 55-like
Pink	B2KJ76	WRKY transcription factor 55-like
Pink	B2KJ84	WRKY transcription factor 65
Pink	B2KKQ8	WRKY transcription factor 67
Pink	F2CVJ7	PHYTOCHROME INTERACTING FACTOR-LIKE 13
Pink	F2DZG5	transcription factor RF2b
Pink	F2E4Q9	WRKY transcription factor 67

Regarding the kinases, results showed that the two significant modules related to barley cold stress response, distinct kinases were annotated in two modules. As presented in Table 4, serine/threonine-protein kinase PBL7, receptor-like protein kinase At2g42960, hexokinase-2, abscisic acid-inducible protein kinase, and bifunctional riboflavin kinase/FMN phosphatase were among the identified kinases in the brown module. Contributions of some other kinases such as CBL-interacting protein kinase 15-like, receptor-like cytoplasmic kinase 176, proline-rich receptor-like protein kinase PERK9 in barley cold stress response were highlighted in the pink module.

**Table 4:** Identified Kinases in two brown and pink modules as a cold response associated modules.

Modules	Uniprot ID	Kinase
Brown	F2CRU6	isopentenyl phosphate kinase
Brown	F2CW03	hexokinase-2
Brown	F2D115	LRR receptor-like serine/threonine-protein kinase At2g16250
Brown	F2D7E2	receptor-like protein kinase At2g42960
Brown	F2D8E2	calcium-dependent protein kinase 8-like
Brown	F2DDR9	hexokinase-5
Brown	F2DGA4	serine/threonine-protein kinase PBL7
Brown	F2DRD7	bifunctional riboflavin kinase/FMN phosphatase
Brown	F2DVP6	casein kinase 1-like protein HD16
Brown	F2DY02	diacylglycerol kinase 5-like
Brown	F2E9Q5	abscisic acid-inducible protein kinase
Brown	F2EA95	cyclin-dependent kinase inhibitor 4-like
Brown	F2EE84	LIM domain-containing serine/threonine-protein kinase DDB_G0286997
Brown	F2EFS9	serine/threonine-protein kinase 24-like
Brown	F4YGN8	hexokinase-2
Brown	Q93VM3	abscisic acid-inducible protein kinase
Pink	F2CSZ0	phosphatidylinositol 4-phosphate 5-kinase 4
Pink	F2CX32	pyruvate kinase 1, cytosolic
Pink	F2D1U7	UMP-CMP kinase 3
Pink	F2D7I9	CBL-interacting protein kinase 15-like
Pink	F2DB51	proline-rich receptor-like protein kinase PERK9
Pink	F2DZT4	wall-associated receptor kinase 4-like
Pink	F2E0Q8	serine/threonine-protein kinase TOUSLED
Pink	F2E6E0	G-type lectin S-receptor-like serine/threonine-protein kinase At2g19130
Pink	Q2EF50	receptor-like cytoplasmic kinase 176

Using the intramodular connectivity criterion, the hub genes which had critical importance and representative of the module's overall function were defined in each significant module. To validate the hub genes discriminative power between control and cold stressed condition, the LOOCV method was applied to the expression value of hub genes as described in Ref. [25]. Results showed that the identified hub genes have distinguished two conditions with 92.01 % accuracy, highlighting the discriminative efficiency of identified hub genes and validating the defined hubs.

### 3.5 TFs- hub-kinase genes regulatory network

**Fig. 4:**
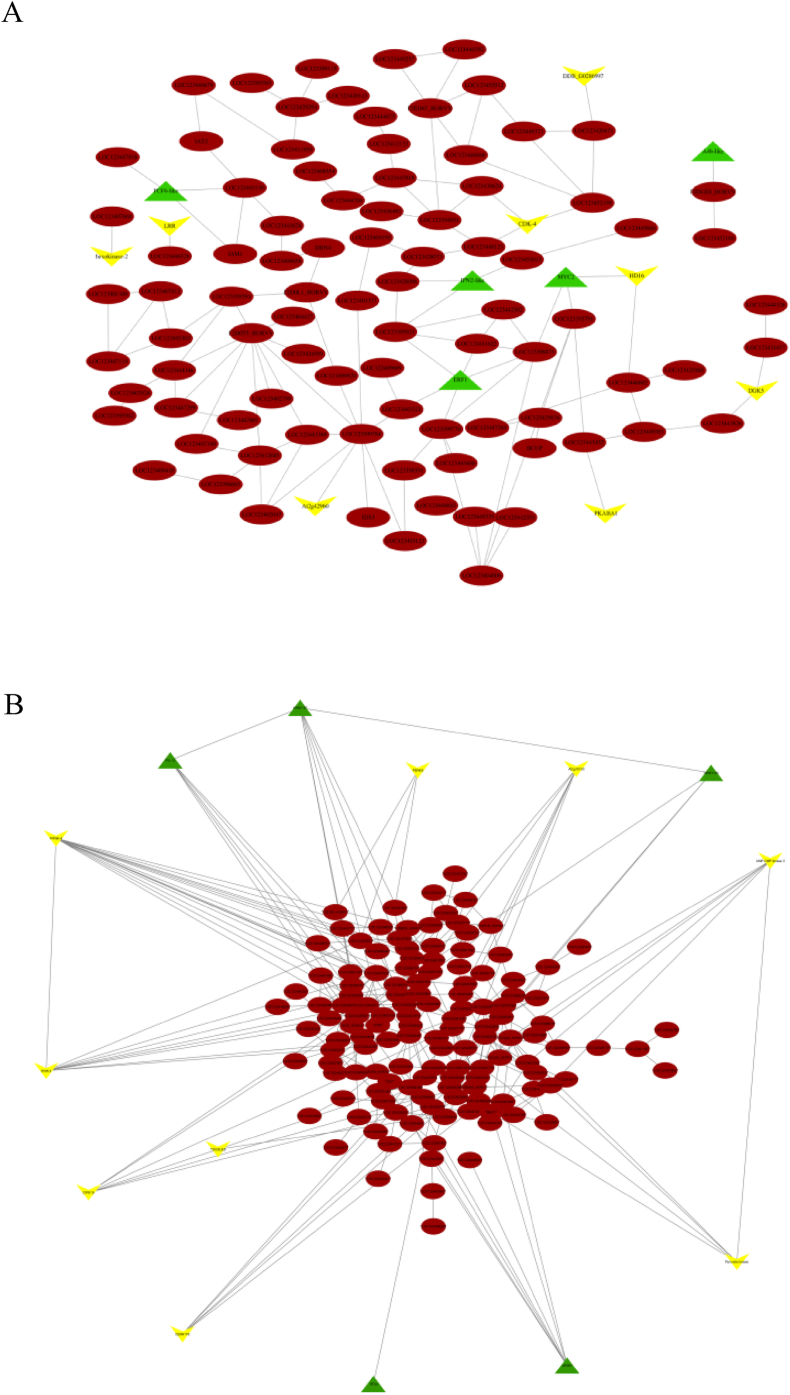
Regulatory networks which were constructed between brown (A) and pink (B) modules, TFs, and Kinases. Circles, triangle, and V shape nodes represent the genes, TFs, and kinases, respectively. (For interpretation of the references to color in this figure legend, the reader is referred to the Web version of this article.)

As mentioned in the methods and material section, the ensemble of tree algorithms with the number of trees = 1000 was harnessed for regulatory network inference between TFs, Kinases, and top hub genes in significant modules (Fig. 4). It was shown that the transcription factor PCF6, ERF1, myb family transcription factor IPN2, and MYC2 regulates the brown module. In the other hand, CDK2, HD16, DGK5, and PKABA1 were examples of the kinases the directly regulate the brown module as depicted in Fig. 4A. Moreover, WRKY-65, WRKY-67, WRKY-55, WRKY-4, RF2b, and PIL13 were examples of kinases which directly interacting in the pink module. PERK9 and CIPK15 kinases which encode the proline-rich extensin-like receptor kinase and Calcineurin B-like interacting protein kinase 15, respectively, were among the interacting kinases in pink module (Fig. 4B).

## 4 Discussion

High-throughput expression profiling approaches provides promising chances for deciphering the different complex processes underlying mechanisms. Given the complex nature of stress-responsive mechanisms, generalization of the cold underlying responses in barley are of central importance to breeding of cold tolerant barley. A considerable amount of literature has been published to uncover the barley cold stress underpinning mechanisms. However, what we know is that most of these studies largely have been focused on differential genes identification and interaction network reforming under cold stress condition has been not mostly focused. The present study was designed to determine the effect of cold stress conditions on the genes behavior at the system level. Moreover, hub genes as important genes and interaction with transcription factors and kinases were investigated using gene co-expression network analysis. The results showed that the topological features of the co-expression network of cold stress-responsive genes changed under cold stress conditions in comparison with control conditions and these genes created some co-expressed modules with distinct functional enrichments (Fig. 4). As mentioned in the results section, two modules including brown and pink were defined as significant modules related to cold stress responses which were considered for further discussion.

Ubiquitin-dependent protein catabolic process was among the most significant enriched process in the brown module. Suh et al. [26] reported that the Arabidopsis RING E3 ubiquitin ligase AtATL80 is negatively involved in phosphate mobilization and cold stress response in sufficient phosphate growth conditions. Moreover, it was shown that the U-box E3 ubiquitin ligase are involved in the positive regulation of low temperature stress response in rice [27]. Results of current study showed that three E3 ubiquitin ligase including E3 ubiquitin-protein ligase SINAT2-like (LOC123404423), E3 ubiquitin-protein ligase AIRP2-like (LOC123429876), and BOI-related E3 ubiquitin-protein ligase 1-like (LOC123404195), regulate the cold stress responses in barley. Other Ubiquitin-proteasome system components which involved in barley cold stress responses were ubiquitin-conjugating enzyme E2 22-like (LOC123411811 and ubiquitin-fold modifier-conjugating enzyme 1 (LOC123449589). Responses of plants to cold stress are mediated by a transcriptional cascade. It was demonstrated that the cold stress induces the degradation of ICE1, which is mediated by HOS1, a RING-type E3 ligase [28]. Accordingly, it was shown that the overexpression of AtCHIP, a U-box-type E3 ligase, renders *Arabidopsis* plants more sensitive to temperature stress [29].

It has been proven that the ubiquitin-proteasome system (UPS) components regulate the ABA biosynthesis or ABA signaling and hence the ABA-dependent regulation of stomatal closure under cold stress condition [30]. Our findings also highlighted the coordinate expression and connectivity between the stomatal closer related genes, ABA activated signaling pathway, and UPS component in barley under cold stress condition (Table 3).

More dissection of the significantly enriched GOs of brown module showed that the proteasome regulation is another important element in the barley responses to cold stress. By modulating the TFs and kinases, the proteasome-mediated protein degradation plays a central role in regulating transcriptional changes required for stress adaption [31]. The contribution of the proteasome-mediated protein degradation in respond to cold stress is reported by different studies. Proteasome subunit alpha type-3 (LOC123399784), 26S proteasome regulatory subunit 6B homolog (LOC123424599), and proteasome subunit alpha type-7-A (LOC123407500) were found in the brown modules. It has been demonstrated that the 26S proteasome is required for hormone signaling pathways mediated by brassinosteroids (BR), ABA, cytokinin (CK), gibberellic acid and jasmonic acid (JA), and suggested that hormones may regulate PTRE1, and hence proteasome activity, to initiate relevant stress responses [32]. Our constructed regulatory network also showed that the proteasome subunit alpha type-3 (LOC123399784) directly regulated by receptor-like protein kinase At2g42960 (Fig. 4) which highlighted the regulating impacts of the mentioned kinase on proteasome regulation under cold stress condition.

Cold stress triggers the plasma membrane rigidification and reforms the cytoskeleton and elevates the cytosolic Ca2^+^ concentration. To modulate the Ca2^+^ concentration of cells, ligand-activated Ca2^+^ channels activated and subsequently induce the mitogen-activated protein kinase (MAPK) signaling pathway and phosphorylation of transcriptional regulators [33]. Moreover, it has been proven that the ROS influence on Ca2^+^ channels to regulate other channels that are influenced by abscisic acid (ABA)-induced stomatal movements under cold stress condition [34]. It has been confirmed that the ROS participate in the cold response through the induction of the cytosolic Ca2^+^ transients and subsequently regulates stomatal closure [35]. In line with the proposed mechanism, dissection of brown modules also showed that there is the coordinated manner in term of the gene expression and connectivity between the most of the Ca2^+^ concentration modulating elements such as CaM-like proteins (CMLs) and CDPKs as Ca2^+^ sensor, calmodulin1, stomatal movements involved genes, ABA, and ROS biosynthesis related genes.

Furthermore, prior researches have proven that ROS activate some of the redox-responsive proteins and ROS detoxification regulating elements such as transcription factors and kinases. In agreement with the mentioned findings, our analysis also highlighted the coordinated regulatory network between ROS detoxification elements and some Kinases such as receptor-like protein kinase At2g42960, calcium-dependent protein kinase 8-like in brown module under cold stress condition.

**Fig. 5:**
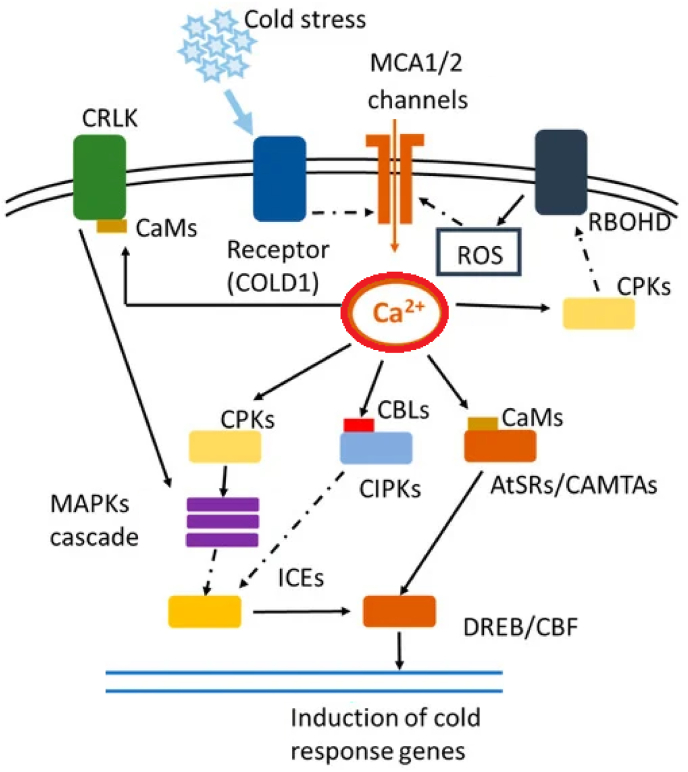
Cross talk between ROS, signaling network and Transcriptional regulatory networks in response to cold stress.

More interestingly the constructed regulatory network based on the TFs, Kinases, and brown module containing genes highlighted co-regulatory impacts of the serine/threonine-protein kinase PBL7, LRR receptor-like serine/threonine-protein kinase At2g16250, serine/threonine-protein kinase 24-like, LIM domain-containing serine/threonine-protein kinase DDB_G0286997, and abscisic acid-inducible protein kinase and transcription factor PCL1-like, transcription factor MYC2, and heat stress transcription factor A-4b-like (Fig. 4A and Table 4). It has been confirmed stress condition leading to activate of the CIPKs by CBLs in the presence of Ca2^+^ is conducted in hormone reactions and ion transport processes (Fig. 5) [36].

Analyses of the significantly enriched GO and KEGG terms revealed that the translation related genes, Cell wall-related pathways, and carbohydrate underlying metabolisms are coordinately regulated and connected in the pink module (Table 2, Table 4). Cell wall-related gene which involved in pectin and cellulose biosynthesis were shown to be up-regulated under cold stress [4]. However our study also highlighted the coordinate connectivity of these genes with carbohydrate underlying metabolisms under cold stress. Prior study also proven that membrane integrity, protoplast properties, and cell wall properties is among the most important factors which affect the plant cold stress tolerance. Our analysis also highlighted the impacts of wall-associated receptor kinase 4 and WRKY transcription factors in expression and connectivity reforming of regulatory networks during barley cold stress condition (Table 4).

As above-mentioned, Ribosome-mediated translation is among the enriched GO and KEGG terms in pink module. It has been demonstrated that the Ribosome-mediated translation inhibits the senses of cold stress and act as a cry sensor and the rate of protein synthesis was found to be proportional decrease in temperature stress condition [37]. Our finding also proposed the contribution of WRKY TFs and CBL-interacting protein kinase 15-like in regulating of Ribosome-mediated translation during cold stress condition (Fig. 4). In line with our finding, it has been demonstrated that this kinase regulates particular responses against cold stresses through modulation of Ca2^+^-binding affinity, expression patterns, subcellular localization, and interaction pattern (Chao et al., 2016).

In conclusion, the systems biology approach which was used in this study revealed the important metabolisms and pathways which expression and connectivity patterns of involved genes were changed under cold stress condition. Furthermore, we delineated the most important hub genes of enriched pathways of significantly related module to construct the regulatory networks with identified TFs and Kinases using machine learning model. Our analysis highlighted the functional importance of ABA signaling pathway, ROS signaling, defensive and protective proteins, degrading protein, Ca2^+^ related signaling, ribosome-mediated translation and etc. in responding of barley to cold stress condition. Moreover, this study provides valuable information regarding the regulatory impacts of different TFs and kinases in different significant models related hubs. The current findings add substantially to our understanding of the cold responsive underlying mechanism of barley which can serve in future studies and breeding programs.

## Data availability

Data was used in current study can be found in GEO with GSE10329, GSE10332, GSE27821, GSE27822 accessions.

## CRediT authorship contribution statement

**Bahman Panahi:** Conceptualization, Formal analysis, Writing – original draft, Writing – review & editing. **Ali Shahi:** Writing – original draft, Writing – review & editing.

## Declaration of competing interest

We declare that there is not any conflict of interest.

## References

[1] Li W., Gao S., Lei T., Jiang L., Duan Y., Zhao Z., Yang L. (2022). Transcriptome analysis revealed a cold stress-responsive transcription factor, PaDREB1A, in Plumbago auriculata that can confer cold tolerance in transgenic Arabidopsis thaliana. Front. Plant Sci..

[2] Hanson H.I., Eckberg E., Widenberg M., Olsson J.A. (2021). Gardens' contribution to people and urban green space. Urban For. Urban Green..

[3] Dumont E., Bahrman N., Goulas E., Valot B., Sellier H., Hilbert J.-L., Vuylsteker C., Lejeune-Hénaut I., Delbreil B. (2011). A proteomic approach to decipher chilling response from cold acclimation in pea (Pisum sativum L.). Plant Sci..

[4] Bray E.A. (2004). Genes commonly regulated by water-deficit stress in Arabidopsis thaliana. J. Exp. Bot..

[5] Panahi B., Mohammadi S.A., Khaksefidi R.E., Mehrabadi J.F., Ebrahimie E. (2015). Genome-wide analysis of alternative splicing events in Hordeum vulgare: highlighting retention of intron-based splicing and its possible function through network analysis. FEBS Lett..

[6] Kasuga Mie, Liu Qiang, Miura Setsuko, Yamaguchi-Shinozaki1 Kazuko, Shinozaki Kazuo (1999). Improving plant drought, salt, and freezing tolerance by gene transfer of a single stress-inducible transcription factor. Nat. Biotechnol..

[7] Chinnusamy V., Schumaker K., Zhu J.K. (2004). Molecular genetic perspectives on cross-talk and specificity in abiotic stress signalling in plants. J. Exp. Bot..

[8] Colcombet J., Hirt H. (2008). *Arabidopsis* MAPKs: a complex signalling network involved in multiple biological processes. Biochem. J..

[9] Min K., Chen K.T., Arora R. (2020). A metabolomics study of ascorbic acid-induced in situ freezing tolerance in spinach (*Spinacia oleracea* L.). Plant Direct.

[10] Mare C., Mazzucotelli E., Crosatti C., Francia E., Stanca A.M., Cattivelli L. (2004). HvWRKY38: a new transcription factor involved in cold- and drought response in barley. Plant Mol. Biol..

[11] Davletova S., Schlauch K., Coutu J., Mittler R. (2005). The zinc-finger protein Zat12 plays a central role in reactive oxygen and abiotic stress signaling in *Arabidopsis*. Plant Physiol..

[12] An J.P., Wang X.F., Zhang X.W., Xu H.F., Bi S.Q., You C.X., Hao Y.J. (2020). An apple MYB transcription factor regulates cold tolerance and anthocyanin accumulation and undergoes MIEL1-mediated degradation. Plant Biotechnol. J..

[13] Shi Y., Ding Y., Yang S. (2015). Cold signal transduction and its interplay with phytohormones during cold acclimation. Plant Cell Physiol..

[14] Yousefi S., Marchese A., Salami S.A., Benny J., Giovino A., Perrone A., Martinelli F. (2022). Identifying conserved genes involved in crop tolerance to cold stress. Funct. Plant Biol..

[15] Panahi B., Mohammadi S.A., Ruzicka K., Abbasi Holaso H., Zare Mehrjerdi M. (2019). Genome-wide identification and co-expression network analysis of nuclear factor-Y in barley revealed potential functions in salt stress. Physiol. Mol. Biol. Plants.

[16] Farhadian M., Rafat S.A., Panahi B., Mayack C. (2021). Weighted gene co-expression network analysis identifies modules and functionally enriched pathways in the lactation process. Sci. Rep..

[17] Panahi B., Farhadian M., Hejazi M.A. (2020). Systems biology approach identifies functional modules and regulatory hubs related to secondary metabolites accumulation after transition from autotrophic to heterotrophic growth condition in microalgae. PLoS One.

[18] Ghahramani N., Shodja J., Rafat S.A., Panahi B., Hasanpur K. (2021). Integrative systems biology analysis elucidates mastitis disease underlying functional modules in dairy cattle. Front. Genet..

[19] Panahi B., Tajaddod S., Mohammadzadeh Jallali H., Hejazi M.A., Zeinalabedini M. (2022). Variability and association among some pomological and physiochemical traits in spring frost tolerant genotypes of Persian walnut (Juglans regia L.) and selection of genotypes with superior traits based on machine learning algorithms. Genet. Resour. Crop Evol..

[20] Daneshafrooz N., Bagherzadeh Cham M., Majidi M., Panahi B. (2022). Identification of potentially functional modules and diagnostic genes related to amyotrophic lateral sclerosis based on the WGCNA and LASSO algorithms. Sci. Rep..

[21] Zheng L., White R.H., Cash V.L., Jack R.F., Dean D.R. (1993). Cysteine desulfurase activity indicates a role for NifS in metallocluster biosynthesis. Proc. Natl. Acad. Sci. USA.

[22] Leek J.T., Johnson W.E., Parker H.S., Jaffe A.E., Storey J.D. (2012). The sva package for removing batch effects and other unwanted variation in high-throughput experiments. Bioinformatics.

[23] Arend M. (2022).

[24] Pérez-Rodríguez P. (2010). PlnTFDB: updated content and new features of the plant transcription factor database. Nucleic Acids Res..

[25] Panahi B., Hejazi M.A. (2021). Weighted gene co-expression network analysis of the salt-responsive transcriptomes reveals novel hub genes in green halophytic microalgae Dunaliella salina. Sci. Rep..

[26] Suh J.Y., Kim W.T. (2015). Arabidopsis RING E3 ubiquitin ligase AtATL80 is negatively involved in phosphate mobilization and cold stress response in sufficient phosphate growth conditions. Biochem. Biophys. Res. Commun..

[27] Byun W.H., Cui L.H., Oh T.K., Jung Y.J., Lee A., Park K.Y., Kim W.T. (2017). Homologous U-box E3 ubiquitin ligase OsPUB2 and OsPUB3 are involved in the positive regulation of low temperature stress response in rice (Oryza sativa L.). Front. Plant Sci..

[28] Dong C., Agarwal M., Zhang Y., Xie Q., Zhu J. (2006).

[29] Luo J., Shen G., Yan J., He C., Zhang H. (2006). AtCHIP functions as an E3 ubiquitin ligase of protein phosphatase 2A subunits and alters plant response to abscisic acid treatment. Plant J..

[30] Dong H.S., Moon Y.R., Fabien J., Jae H.H., Michelle T., Bin G.K., Woo T.K. (2012). Roles of four Arabidopsis U-Box E3 ubiquitin ligases in negative regulation of abscisic acid-mediated drought stress responses. Plant Physiol..

[31] Xu F.Q., Xue H.W. (2019). The ubiquitin‐proteasome system in plant responses to environments. Plant Cell Environ..

[32] Kelley D.R., Estelle M. (2012). Ubiquitin-mediated control of plant hormone signaling. Plant Physiol..

[33] Conde A., Chaves M.M., Geros H. (2011). Membrane transport, sensing and signaling in plant adaptation to environmental stress. Plant Cell Physiol..

[34] Cho J.H., Lee J.H., Park Y.K., Choi M.N., Kim K.N. (2016). CalcineurinB-like protein CBL10 directly interacts with TOC34 (translocon of the outer membrane of the chloroplasts) and decreases its GTPase activity in Arabidopsis. Front. Plant Sci..

[35] Wang W.H., He E.M., Guo Y., Tonga Q.X., Zheng H.L. (2016). Chloroplast calcium and ROS signaling networks potentially facilitate the primed state for stomatal closure under multiple stresses. Environ. Exp. Bot..

[36] Li Z.Y., Xu Z.S., Chen Y., He G.Y., Yang G.X., Chen M., Li L.C., Ma Y.Z. (2013). A novel role for Arabidopsis CBL1 in affecting plant responses to glucose and gibberellin during germination and seedling development. PLoS One.

[37] Guillaume-Schöpfer D., Jaeger K.E., Geng F., Doccula F.G., Costa A., Webb A.A., Wigge P.A. (2020).

